# Supplementary material for: Very-large-scale reconfigurable intelligent surfaces for dynamic control of terahertz and millimeter waves
Source: Nat Commun. 2025 Mar 25;16:2907. doi: 10.1038/s41467-025-58256-w (PMC11937571; doi:10.1038/s41467-025-58256-w)
Supplement: Supplementary file 1 — Supplementary Information [file 41467_2025_58256_MOESM1_ESM.pdf]

Supporting Materials for

"Very-Large-Scale Reconfigurable Intelligent Surfaces for Dynamic Control of Terahertz and Millimetre Waves"

Yury Malevich[1,2], M. Said Ergoktas[1,2,3], Gokhan Bakan[1,2], Pietro Steiner[1,2]
Coskun Kocabas[1,2,4,*]

[1]Department of Materials, University of Manchester, Manchester, M13 9PL, UK

[2]National Graphene Institute, University of Manchester, Manchester, M13 9PL, UK

[3] Department of Physics, University of Bath Claverton Down, BA2 7AY, UK

[4]Henry Royce Institute for Advanced Materials, University of Manchester, Manchester M13 9PL, UK

*Corresponding authors E-mail: coskun.kocabas@manchester.ac.uk

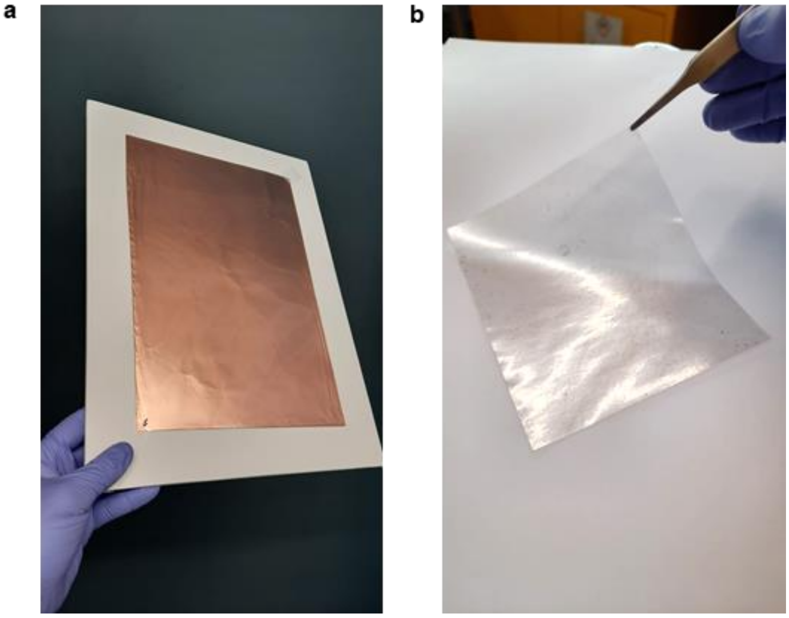

Figure S1: **a**, Large-area graphene on copper foils grown by chemical vapour deposition. A4 size samples were obtained from MCK Tech (S. Korea). B, Double layer graphene on PET polymer sheets obtained by sequential transfer process.

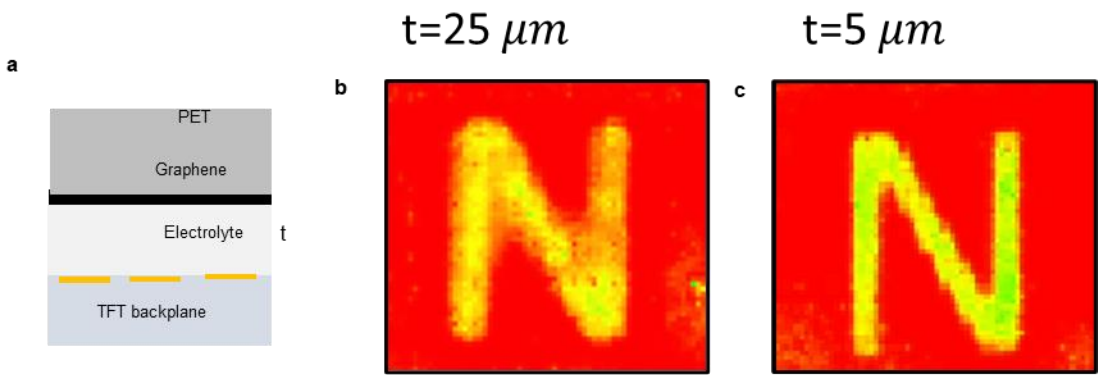

Figure S2: Effect of electrolyte thickness: **a**, Cross sectional view of the device showing TFT backplane, the electrolyte layer containing ionic liquid (DEME TFSI) and porous polyethylene membrane, and top polymer layer. **b, c** shows the transmission images obtained from two different devices with 25 and 5μm electrolyte thickness. The electrolyte thickness determines the crosstalk between the pixels.

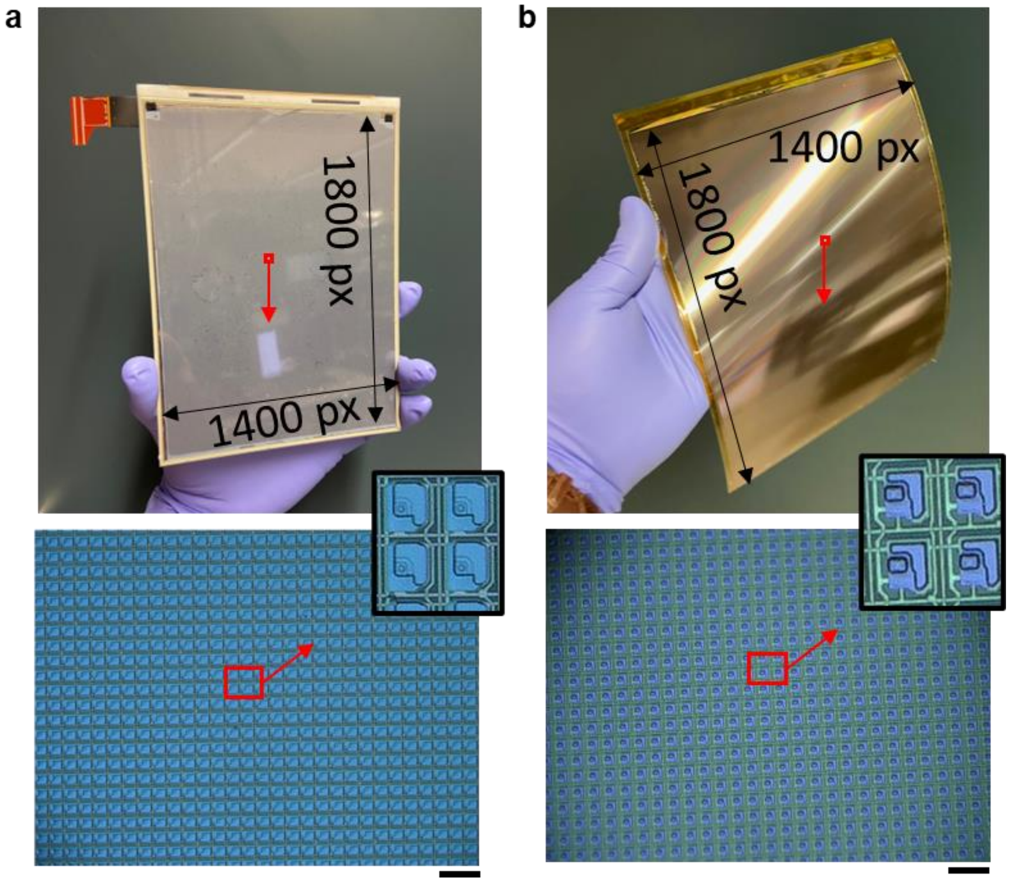

Figure S3: Megapixel THz modulator arrays: Photograph of 2.5 megapixel (1800x1400) of TFT arrays on glass (**a**) and flexible Kapton (**b**) substrates. The pixel size is 0.11x0.11 mm. The scale bar shows 0.3 mm. The inset shows the individual pixels and complex wiring. The scalability of our approach is primarily constrained by the capabilities of modern display technology, which enables the production of very large-scale, dense arrays of TFTs on both rigid and flexible substrates. In the manuscript, we only characterized a 0.3-megapixel TFT device. However, our method is capable of scaling the device size to multiple megapixels with suitable back plane electronics.

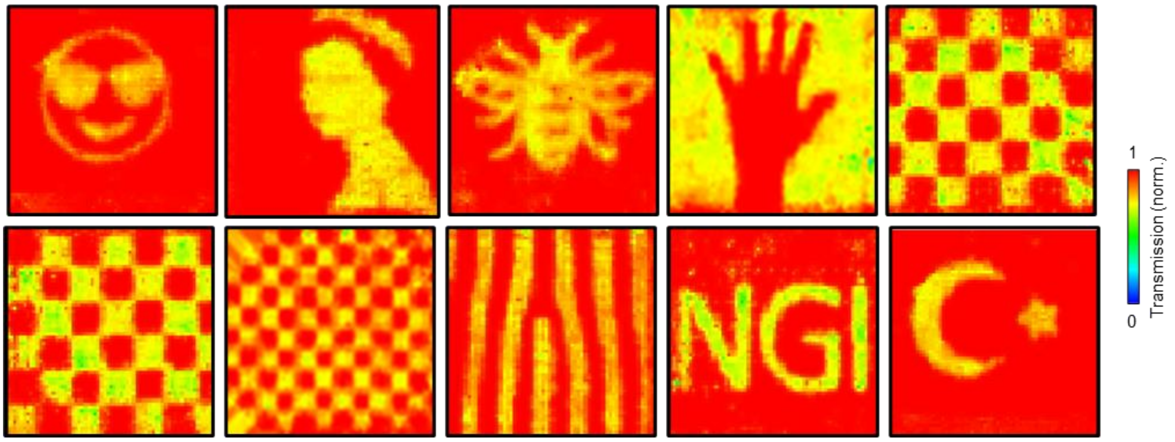

Figure S4, Various transmission images generated by the modulator at 0.1 THz. These images were recorded by Terasense 64x64 pixel camera.

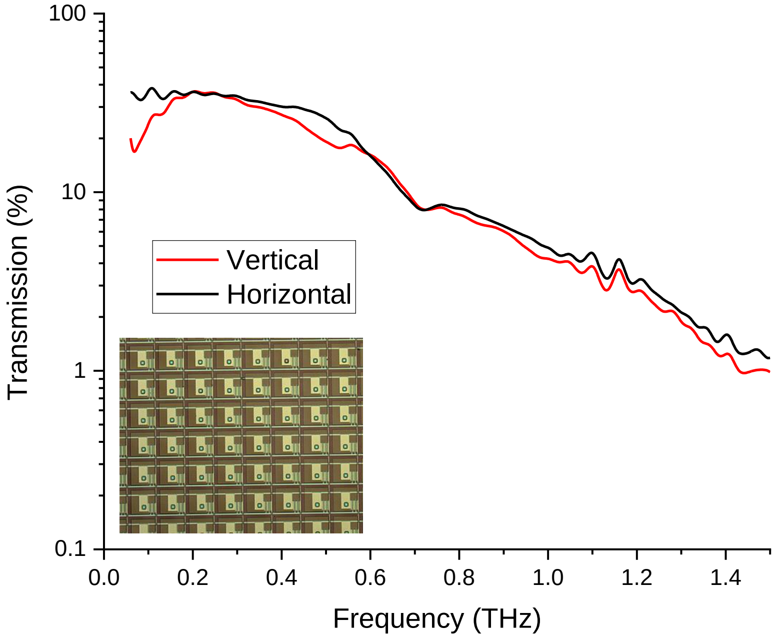

Figure S5: *Transmission spectrum of the modulator array. The inset shows the picture of the TFT array to provide reference for the direction of polarization.* The transmission measurements presented in the paper were recorded using 100 GHz source with horizontal polarization.

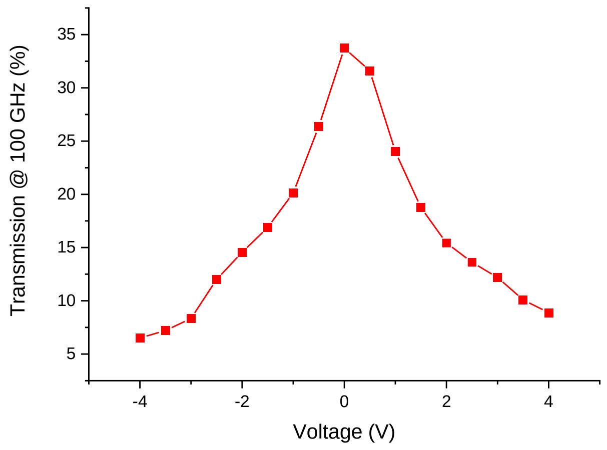

Figure S6: *Voltage dependent transmission of double layer graphene modulator shown in Figure 2. This curve is obtained at static mode, by applying external voltage to the graphene layers as the pixel electrodes are grounded. It should be noted that the effective voltage during the operation of the modulator depends on many factors such as refresh rate of the modulator, drain voltage and pulse duration. Therefore, drain voltage and external voltage are not the same quantity.*

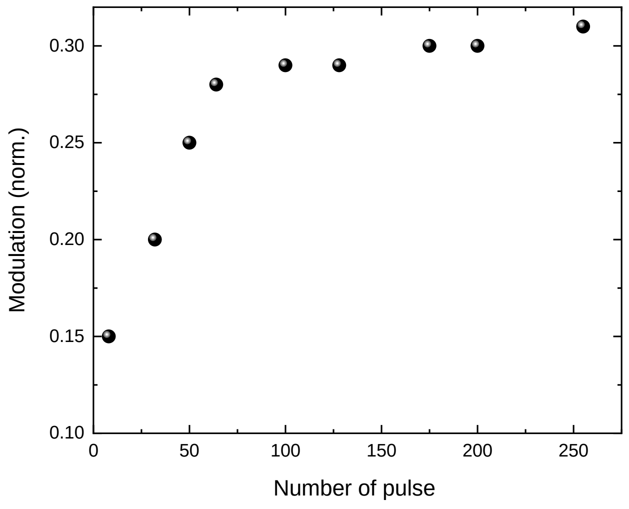

Figure S7: *The variation of the transmission modulation as a function of the number of applied pulses to the pixel. Here the pulse width is 30 µsec. The increasing number of pulses increases the modulation depth, however it reduces the total time to refresh the pattern on the modulator.*

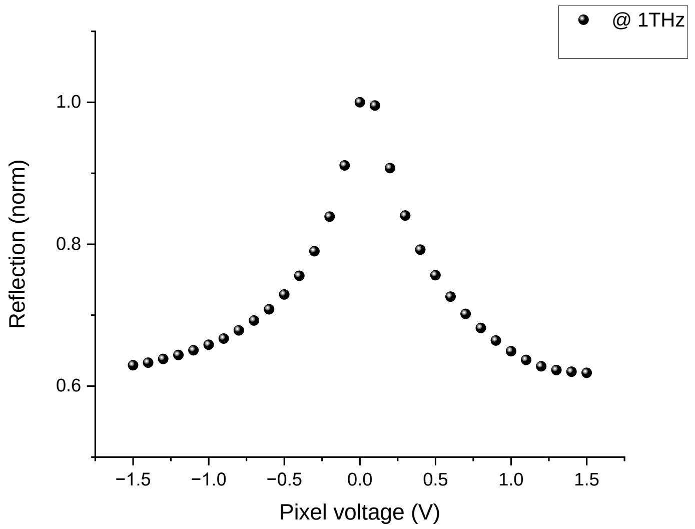

***Figure S8**: The variation of the reflection at 1THz as a function of pixel voltage. It should be noted this this measurement obtain in the static mode by grounding the electrodes and changing the voltage applied to the graphene. Due to the complexity of the control electronics and the active-matrix circuit, the drain voltage does not appear directly on the pixel due to charging time.*

Numerical Simulation of the charging dynamics:

To understand the charge dynamics and speed of the modulator, we have performed Finite Element Method (FEM) simulations based on Poisson–Nernst–Plank (PNP) equations similar to reference 29-31 . To capture the dynamics of IL electrolyte in PNP equations which can only capture the drift-diffusion dynamics of dilute electrolyte, we have included finite size effects and diffusion process of ionic liquids of ions which requires precise modifications of the differential equations. We have threated the room temperature IL as a solvent free, molten salt which maintains uniform total ion density ($p + n = 2c_0$) where n and p are the density of anion and cations and we set the initial ion concentration to $2c_0 \cong 3.3\ M\ and$ through the ionic steric interactions maximum $c_0 \cong 6.6\ M$. Since there is no solvent in the RIL, the diffusion dynamics of IL is driven by inter-diffusion process which can be implemented by following the Onsager' framework ($\mathbf{J} \sim pn\nabla(\mu)$), where is μ the chemical potential.

We restrict our model to Chan-Hillard-Poisson type formalism to include all the steric effects, Columbic interactions and Onsager' diffusion process. Using COMSOL Multiphysics

tool, and using the framework developed by Gavish and Yochelis, the free energy of the electrolyte system can be written as

$$\mathcal{F} = \int k_B T[p \ln p + n \ln n] + \left[q(p-n)\phi - \frac{\epsilon}{2}|\nabla\phi|^2\right] + \frac{1}{c_0}\left[\frac{\beta np}{4} + \frac{\varepsilon_0 \kappa^2}{4}(|\nabla p|^2 + |\nabla n|^2)\right] \qquad (1)$$

p and n are cation and anion molar concentrations, $c_0 = 3.3\,M$ is the initial bulk ion concentration and ϕ is the electric potential. Further, the modified Poisson–Nernst–Plank equations and the corresponding currents are given below;

$$\frac{\partial p}{\partial t} = -\nabla.J \qquad (2)$$

$$\mathbf{J} = \frac{Dpn}{k_B T c_0}\nabla\left(k_B T \ln\frac{p}{n} + 2ze\phi - \frac{\beta(c_{ref}-p)}{2c_0} - \varepsilon_0\kappa^2\nabla^2 p\right) \qquad (3)$$

$$\epsilon\nabla^2\phi = -\,q(p-n) \qquad (4)$$

where, $D_i \cong 1x10^{-11} m^2/s$ is the isotropic diffusion coefficient, q is the elementary charge and $\epsilon{\sim}20$ is dielectric constant of the IL We assume equal ion diameter $a \cong 0.4 - 0.6\,nm$ which sets the maximum ion concentration to $2c_0 \cong 6.6\,M$ through the ionic steric interactions.

To validate the numerical simulations, we first focused on 1D parallel plate capacitor configuration. As an initial step we assessed the time response of the capacitor and contribution of steric effects and clustering dynamics to this charging process. We transiently applied voltage step(1V) to one electrode and grounded the other one. Figure S4 summarizes the time responses of the IL on the pixelated surface. Although the initial voltage distribution across the pixel is linear between 0 to 1V, at the steady state, through the two equivalent EDL formation it drops the voltage to the equilibrium level of 0.5 V. As ions accumulate on the electrodes, this initially linear voltage distribution converges to a flat equilibrium profile. Depending on the analysed points on the device the local voltage shows up and down shift towards this equilibrium level. Note that, the center of the device shows flat response, this is because this point is already at the equilibrium condition. As a second step we probed the time response of the system. The results of the simulations are depicted in Figure S4.

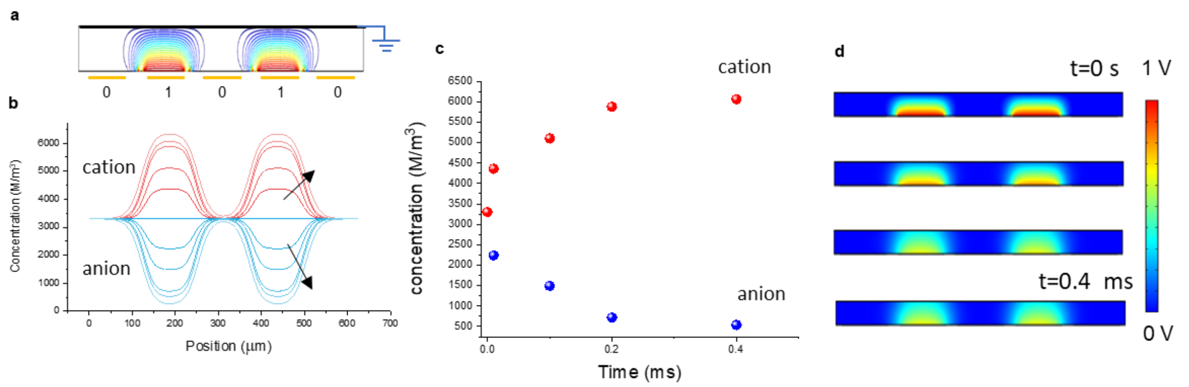

Figure S9: **a**, Cross sectional view of the device and electric field distribution of the pixelated surface with alternating voltages of 0 and 1V right after the voltage is applied. **b**, Spatial distribution of anion and cation concentration on the graphene electrolyte interface at different time intervals after the voltage is applied. Arrows shows the increasing time. **C**, shows the accumulation of cations and depletion of anions on the pixel after the voltage is applied. D shows the spatial voltage distribution across the electrolyte layer. at t=0, the voltage drop shows a linear dependence across the electrolyte, after 0.4 ms, ions in the electrolyte screen the potential and provides electrical-double layer formation resulting a sharp voltage drop at the interface between graphene-electrolyte and electrolyte-electrode.

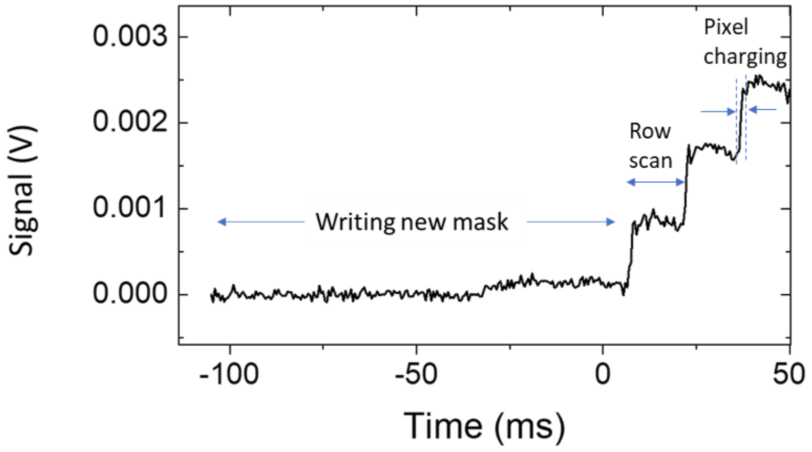

Figure S10: Time trace of the transmitted signal during the pattern update. The speed of single-pixel imaging depends on various factors, including the refresh rate of the TFT array, the communication speed of the driver chip, and the inherent speed of the graphene modulators. The following figure illustrates these timings. The slowest step is data transmission to the driver chip, which takes around 100 ms. After updating the pattern on the modulator, the scan time of the TFT array is between 5–20 ms. The pixel charging time is approximately 1–2 ms. The pattern on the modulator can be refreshed at a rate of 5 frames per second. For the single-pixel images shown in Figure 4, we used 1024 masks, which took around 5 minutes to complete. The main limiting factor for imaging is the processing capacity of the display driver chip. The imaging speed can be increased by using more powerful display drivers.

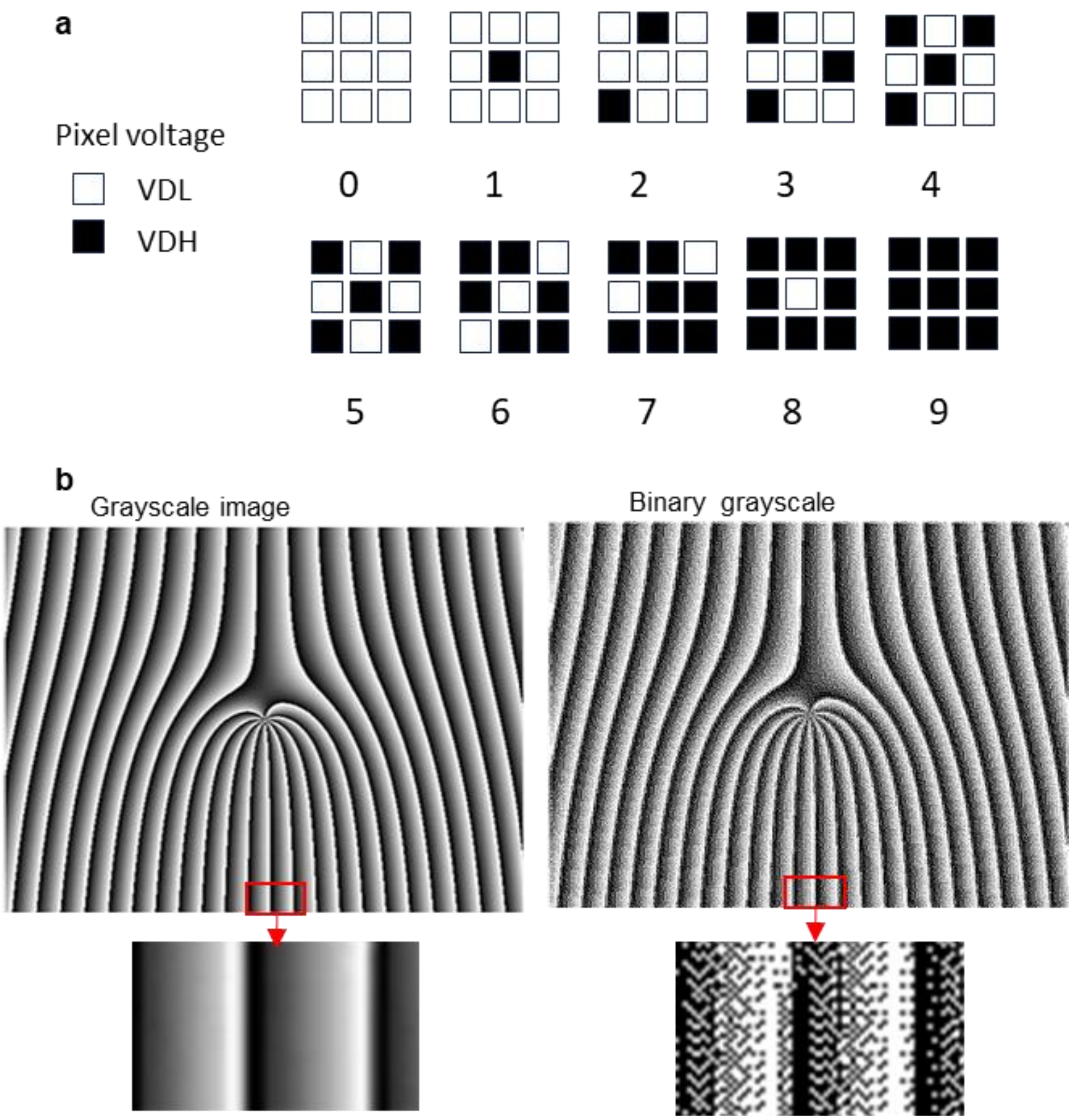

Figure S11: Generation of binary grayscale images. **a**, Supercell consisting of 3x3 array of pixels with binary voltages, V_{DH} and V_{DL}. 10 level grayscale is generated by changing the ratio of the number of pixels with voltage of V_{DH} and V_{DL}. **b**, Grayscale and binary grayscale image of a fork grating with topological charge of q=9.

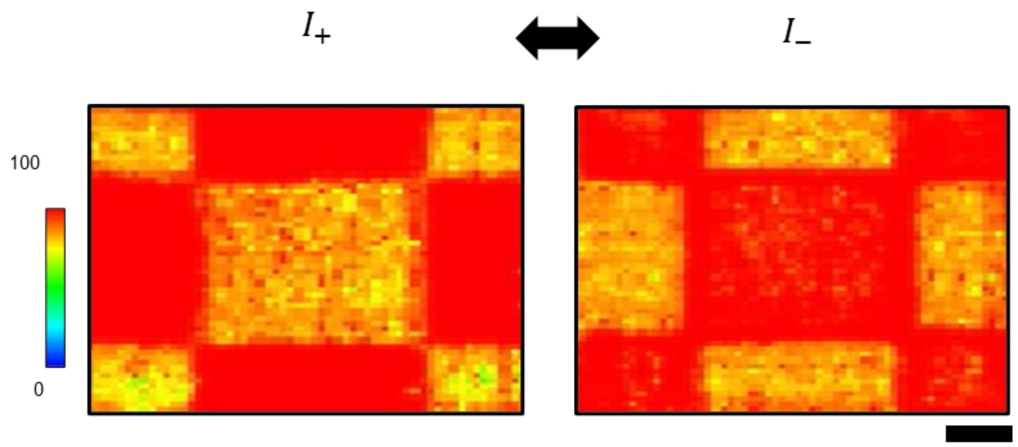

Figure S12: The images of the complementary Hadamard mask generated by changing the VDH and VDL applied to the pixels. The differential signal of the transmitted intensity is used to reconstruct the image.

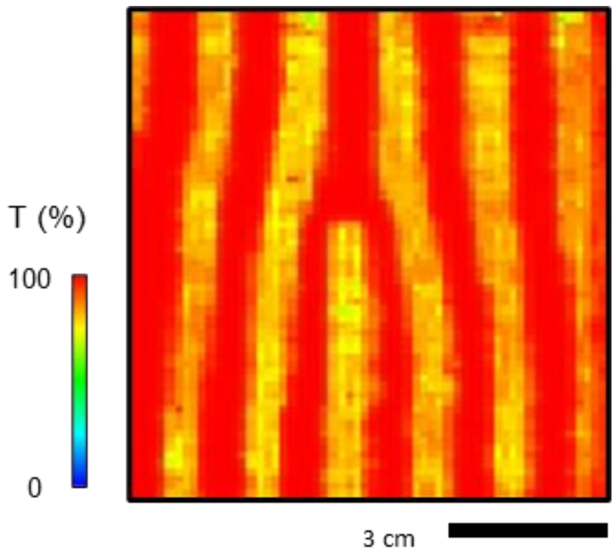

Figure S13: Transmission image of the fork grating with topological charge of 1 generated by the modulator array . The fork grating is obtained by combination of linear grating and radial gradient. This pattern generates a dislocation with a topological charge.
